# Co-morbidities of mental disorders and chronic physical diseases in developing and emerging countries: a meta-analysis

**DOI:** 10.1186/s12889-019-6623-6

**Published:** 2019-03-13

**Authors:** Labanté Outcha Daré, Pierre-Emile Bruand, Daniel Gérard, Benoît Marin, Valerie Lameyre, Farid Boumédiène, Pierre-Marie Preux

**Affiliations:** 1 INSERM, Univ. Limoges, CHU Limoges, UMR_S 1094, Tropical Neuroepidemiology, Institute of Neuroepidemiology and Tropical Neurology, CNRS FR 3503 GEIST, F-87000 Limoges, France; 2 Access to Medicines, SANOFI, SAG / CSVB, 82 AV Raspail, 94250 Gentilly, France

**Keywords:** Meta-analysis, Association, Comorbidities, Mental disorders, Chronic diseases

## Abstract

**Background:**

As the data on the association of mental disorders and chronic physical diseases in developing and emerging countries is heterogeneous, this study aims to produce the first meta-analysis of these comorbidities.

**Methodology:**

The meta-analysis protocol was registered in PROSPERO (N°CRD42017056521) and was performed in accordance with PRISMA guidelines. Initially, an article search was conducted on Medline, Embase, Lilacs and the Institut d’Epidémiologie et de Neurologie Tropicale database [Institute of Epidemiology and Tropical Neurology], as well as manually, with no restriction on language or date focusing on mental disorders, chronic diseases and neurotropic diseases. Two independent investigators assessed the quality of the studies which met the inclusion criteria using the Downs and Black assessment grid. The pooled estimates were calculated out using a random-effects method with CMA software Version 3.0. A meta-regression was then performed, and the significance level was set at 0.05.

**Results:**

Of the 2604 articles identified, 40 articles involving 21,747 subjects met the inclusion criteria for co-morbidities between mental disorders and chronic physical diseases. Thirty-one articles were included in the meta-analysis of prevalence studies and 9 articles in that of the analytical studies. The pooled prevalence of mental disorders in patients with chronic physical diseases was 36.6% (95% CI, 31.4–42.1) and the pooled odds ratio was 3.1 (95% CI, 1.7–5.2). There was heterogeneity in all the estimates and in some cases, this was explained by the quality of the studies.

**Conclusion:**

Some estimates regarding the prevalence of mental disorders in people with chronic physical diseases living in developing and emerging countries were similar to those in developed countries. Mental disorders are a burden in these countries. In order to respond effectively and efficiently to the morbidity and mortality associated with them, mental health care could be integrated with physical care.

**Electronic supplementary material:**

The online version of this article (10.1186/s12889-019-6623-6) contains supplementary material, which is available to authorized users.

## Background

Worldwide, the burden of chronic physical diseases and mental disorders is becoming ever more significant [1, 2]. This increase is largely attributable to the current epidemiological transition in many developing and emerging countries with an ongoing increase in non-communicable diseases in adults [3].

Mental disorders, an essential component of these diseases, are a burden with a huge impact in terms of disability. In 2010, mental disorders and drug addiction accounted for 183.9 million Disability-Adjusted Life Years (DALYs), i.e., 7.4% (6.2–8.6) of all DALYs worldwide [4]. However, the most recent data published by Vigo et al. in 2016 [5], suggest that the global burden of mental illness would actually account for 32.4% of Years Lived with Disability (YLDs), and for 13.0% of DALYs. In this crucial period of epidemiological transition, when developing and emerging countries are facing new health challenges, the studying of co-morbidities for chronic physical diseases is relevant because they represent the greatest cause of death in the world [6]. Mental disorders rank third among the most frequent diseases after cancer and cardiovascular diseases [7]. According to surveys conducted in developed and developing countries, more than 25.0% of individuals have one or more mental or behavioural disorders during their lifetime [8]. In the general population, severe characterized depression affects 3.0% of people, generalized anxiety disorder affects 2.0% and the prevalence of schizophrenia is expressed at nearly 1.0% [9]. In terms of chronic physical diseases, the world’s leading causes of death, it was estimated in 2008 that 36 million deaths were due to chronic physical diseases [10], including 30.0% to cardiovascular diseases, 13.0% to cancers, 7.0% to chronic respiratory diseases, 2.0% to diabetes and 9.0% to other chronic diseases [11, 12].

This situation is even more worrying as mental disorders are often associated with one or more chronic physical diseases and lead to even more aggravating physical consequences for patient health. In addition, the care provided to people with mental disorders for their physical health problems is inferior to that received by persons without mental disorders. The figures indicate that in low- and middle-income countries approximately 80% of people with severe mental disorders have a problem accessing mental health care and do not receive treatment; this makes mental disorders the second highest cause of morbidity and mortality in these countries. The association between mental disorders (depression, anxiety, schizophrenia and bipolar disorders) and chronic physical diseases [13] such as cancer [14, 15], heart disease, stroke [15, 16], diabetes [17–20]), obesity [21] and chronic obstructive pulmonary disease (COPD) [22, 23] has been the subject of several studies. The data on these comorbidities is very heterogeneous in developing and emerging countries (all non-high income countries by World Bank rankings [24]). For some of these comorbidities, the prevalence rates range from 19.0 [25] to 68.1% [26] and some authors assert that these comorbidities are underestimated [27, 28] in these countries. There is, to date, no meta-analysis covering all the developing and emerging countries, and as such, a study might have a significant impact on public health guidelines for these countries; hence the reason why we decided to perform a meta-analysis of the association of mental disorders and chronic physical diseases.

## Purpose of the study

The purpose of this study was to estimate the pooled prevalences and pooled association measures of thevarious associations of mental disorders and chronic physical diseases in developing and emerging countries.

## Methods

### Implementation procedure

When performing this meta-analysis, the diseases of interest were: - for mental disorders: anxiety, depression, bipolar disorder and schizophrenia [5, 29]. Mental health disorders are medical conditions that can interfere with thinking, feeling, mood, a person’s ability to communicate with others and daily functioning. They are medical conditions that often result in a reduced ability to cope with routine daily activities: such as going to work or raising a family [13]. - for chronic physical diseases: diabetes, obesity, cancer, heart disease and COPD [6, 30]. Chronic diseases are non-communicable diseases, prolonged in time, which do not resolve spontaneously, and which are rarely completely curable [5].

The meta-analysis was performed according to Preferred Reporting Items for Systematic Review and Meta-Analysis (PRISMA) guidelines [31], and the method for the meta-analysis of observational studies in epidemiology [32]. Its protocol was registered in PROSPERO on 10 February 2017, under number CRD42017056521 (http://www.crd.york.ac.uk/PROSPERO).

### Research strategy

Initially, this research focused on both the associations between mental disorders and chronic physical diseases and the association between mental disorders and neurotropic parasites. This last type of association will be presented in a second article. A principal investigator (LOD) performed the article search on Medline, Embase, Lilacs and IENT database (the database of the Institut d’Epidémiologie et de Neurologie Tropicale of the Université de Limoges [University of Limoges] http://www-ient.unilim.fr/), and manually, from 11 February to 07 May 2017, with no restriction on language or date. The research equation was constructed on PubMed by country as follows: *(“Depressive Disorder”[Mesh] OR “Depression” [Mesh] OR “Anxiety Disorders”[Mesh] OR “Anxiety” [Mesh] OR “Bipolar Disorder”[Mesh] OR “Schizophrenia”[Mesh****]****)* ***AND*** *(“Diabetes metillus”[Mesh] OR “Obesity”[Mesh] OR “Neoplasms”[Mesh] OR “Cardiovascular Diseases”[Mesh] OR “Pulmonary Disease, Chronic Obstructive”[Mesh]* ***OR*** *“Malaria”[Mesh] OR “Cysticercosis”[Mesh] OR “Toxoplasmosis”[Mesh] OR “Toxocariasis”[Mesh] OR “Trypanosomiasis”[Mesh] OR “Chagas Disease”[Mesh])* ***AND*** *(“Name of a country”[Mesh]).*

In Lilacs and Embase, the same keywords were used in free texts to reconstruct the search equation. And in total, 139 different equations corresponding to 139 countries classified as developing and emerging countries according to the World Bank [24] were set-up on each database (Medline, Embase and Lilacs). In the IENT database, articles were searched for free text by entering the term “comorbidity” or “comorbidity in mental health”. This procedure was adopted because the IENT database is specific to developing and emerging countries. The management (registration and selection) of articles was carried out using Zotéro bibliographic management software.

### Inclusion criteria and selection

To be included in the meta-analysis, each article, which was selected on the basis of its title and then of its summary, had to: be an original article whose full text was available; be a cross-sectional or analytical study; have been conducted on adult patients, both males and females, and on all age groups (age ≥ 15 years); be a study involving either only hospitalised subjects or only non-hospitalised subjects, but not both hospitalised and non-hospitalised subjects at the same time; specify the method of disease diagnosis. For cross-sectional studies, it had to give the prevalence, or the data from which it could be calculated, and for analytical studies, it had to give the association measures or the data from which they could be calculated.

In case of articles that met the inclusion criteria but were published several times by the same authors, with the same main results, only the most recently published article was included. By “non-hospitalized” we mean patients who live in community or in other words who attended to the health centre (clinic or hospital) for care but who did not stay in the health care centre for one or more nights. We chose to name them this way as opposed to “hospitalized”, that is, patients who were staying in a health care centre.

### Data extraction and assessment of article quality

For each article in our study, the principal investigator (LOD) recorded the reference, title, country and continent, the study type and study population, the original disease, the associated disease searched for, and its method of diagnosis, the prevalence or measures of association of the disease searched for (or the data to calculate them), the sample size, sex ratio, mean subject age. The assessment of study quality was performed independently by two investigators (LOD and PEB) using the revised assessment grid of Downs and Black. Each article was assigned a score independently [33, 34] by each investigator and the final score was decided by mutual agreement after comparing the ratings. In the event of a disagreement, the expert opinion of a third investigator (PMP) was requested, without this investigator being aware of the judgments of the first two investigators. The final score was then assigned by mutual agreement by the three investigators.

### Statistical analyses

CMA (Comprehensive Meta Analysis) software Version 3.0 [35] was used for the statistical analyses. Firstly, the potential heterogeneity amongst the selected studies was assessed using statistical [36, 37] Q and *I*^*2*^ tests (*I*^*2*^ = 0, lack of heterogeneity; *I*^*2*^ < 0.25, low heterogeneity; *I*^*2*^ between 0.25 and 0.5, moderate heterogeneity; and *I*^*2*^ > 0.5, significant heterogeneity). The pooled estimates were then calculated using the DerSimonian-Laird random-effects method [38] and the results obtained were presented on a Forest Plot [39] with a significance threshold of 5%.

The investigation for publication bias was performed by constructing a Funnel Plot, the Duval and Tweedie trim and fill test [38] and Egger’s linear regression [40]. To assess the robustness of the principal results of our meta-analysis, we carried out a sensitivity analysis by removing the study with the greatest weighting and the studies of lower quality in the pooled studies. The following variables were used to perform subgroup analyses: type of original disease (type of disease a person had before the study) and type of associated diseases (type of diseases investigated through the study), type of included subjects (hospitalised or non-hospitalised) and continent where the study had taken place. Finally, a meta-regression was performed with the study country income level and the quality scores of studies as covariates.

## Results

**Fig. 1:**
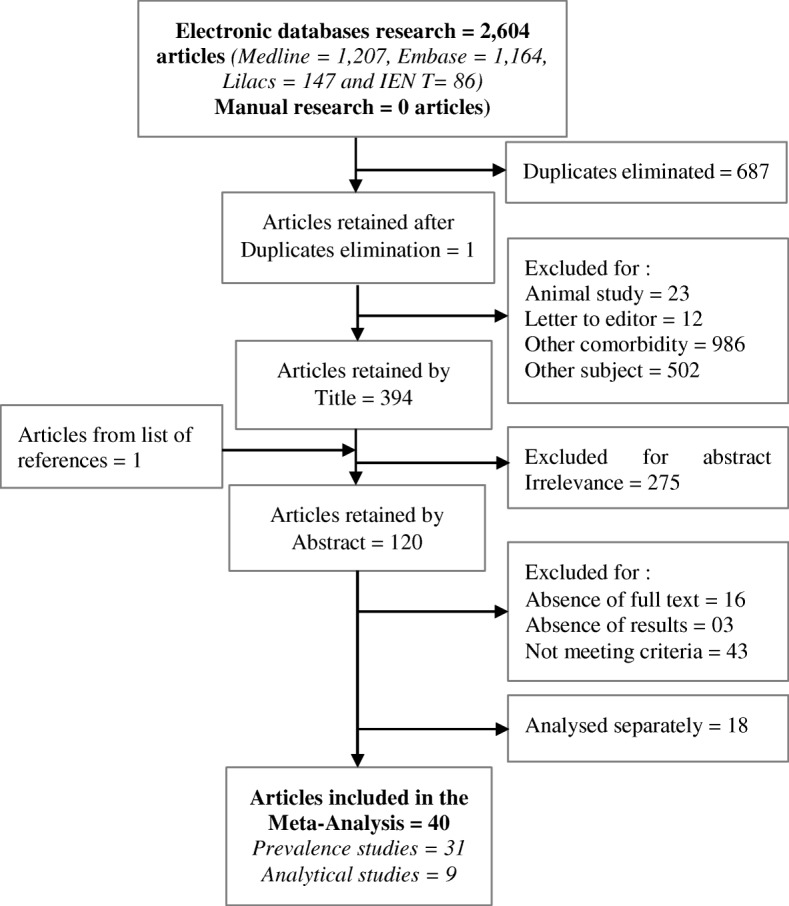
Flow chart of research strategy of the meta-analysis of comorbidities of mental disorders and chronic physical diseases in developing and emerging countries

In total, 2604 articles were found in six languages (English, Spanish, Portuguese, French, Chinese and Russian). After the selection process, 40 articles involving 21,747 participants [41–80] were included in the meta-analysis (Fig. 1) of which 31 articles were prevalence studies [42–46, 48, 50–54, 57, 59–61, 63–65, 67–73, 75–80] and 9 articles were case-control studies [41, 47, 49, 55, 56, 58, 62, 66, 74] (Additional file 1: Table S1 and Additional file 2: Table S2). These articles had been published between 1993 and 2016.

The mean age of the subjects in the prevalence studies was 55.6 ± 11.7 years with a range of [15.0–99.0 years] and the female/male sex ratio was 1.0. In the analytical case-control studies, the mean age of the subjects for the cases was 46.2.0 ± 11.6 years and 42.3 ± 10.4 years for the controls with a range of [16.0–80.0] years for the two groups. The female/male sex ratio was 0.7 for the case-control studies. The subjects enrolled were hospitalised patients in 13 prevalence studies [46, 48, 50, 53, 54, 57, 64, 67, 71, 76, 78–80] and 3 analytical studies [47, 49, 74]. Non-hospitalised subjects had been studied in 18 prevalence studies [42–45, 51, 52, 59–61, 63, 65, 68–70, 72, 73, 75, 77] and 6 analytical studies [41, 55, 56, 58, 62, 66].

Three of the 40 studies had been conducted amongst people with mental disorders who had been screened for chronic physical diseases; two of these were prevalence studies [52, 75] and one was an analytical study [74]. Thirty seven studies had been conducted amongst people with chronic physical diseases who were screened for mental disorders; twenty nine of these were prevalence studies [42–46, 48, 50, 51, 53, 54, 57, 59–61, 63–65, 67–73, 76–80] and 8 were case-control studies [41, 47, 49, 55, 56, 58, 62, 66]. There was one multicentre study conducted in Asia amongst the prevalence studies [70] which was considered in our meta-analysis. In total, by continent, we found 1 study in Europe, 4 studies in Africa, 7 studies in America and 46 studies in Asia.

### Quality of the studies

The quality scores of the studies from the Downs and Black assessment grid were a mean of 16.2 ± 3.2 out of a maximum of 22 for the prevalence studies and a mean of 15.3 ± 3.2 out of a maximum score of 25 for the case-control studies (Additional file 3: Table S3 and Additional file 4: Table S4).

### Pooled estimates and heterogeneity

The diseases found in the prevalence studies were: anxiety and depression for mental disorders; diabetes, obesity, cancer, chronic obstructive pulmonary disease (COPD) and heart disease for the chronic physical diseases. There were 16,462 subjects enrolled in the prevalence studies.

**Fig. 2:**
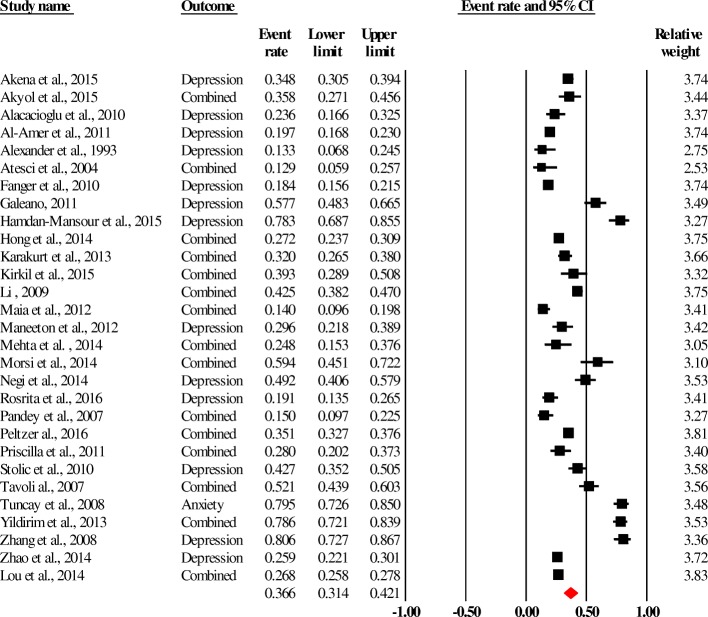
Forest plot of the pooled prevalence of anxiety and / or depression in people with chronic physical illnesses (diabetes, obesity, cancers, COPD and heart disease). Heterogeneity: Q = 795.0, df = 28, *p* < 0.0001, I2 = 96.48**.** Combined = anxiety & Depression

The pooled prevalence of anxiety and/or depression could be estimated from twenty-nine studies involving 16,108 people with chronic diseases (diabetes, obesity, cancer, COPD and heart disease). This pooled prevalence was 36.6% (95% CI, 31.4–42.1) as is demonstrated by the forest plot in Fig. 2. There was heterogeneity in the pooled estimates (Q: 795.01, df: 28; *p* < 0.0001 and *I*^*2*^: 96.48). And, the prevalence of diabetes and/or obesity in the 409 people with mental disorders (schizophrenia and/or depression) was assessed as 16.2% (95% CI, 7.2–32.5).

**Fig. 3:**
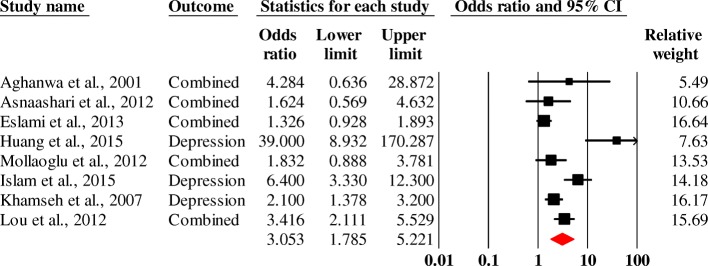
Forest plot of the pooled odds ratio of anxiety and / or depression in people with chronic physical illness (diabetes, obesity, COPD and heart disease).Heterogeneity: Q = 36.81, df = 7, p < 0.0001, I2 = 80.96

The mental disorders found included anxiety, depression, schizophrenia and bipolar disorder. The chronic physical diseases found were chronic coronary heart disease, diabetes, obesity and COPD. With 8 case-control studies (2448 people with chronic diseases and 2422 controls), a pooled odds ratio of 3.1 (95% CI, 1.8–5.2) (Fig. 3) was found for anxiety and/or depression in people with chronic physical diseases.

### Publication bias

**Fig. 4:**
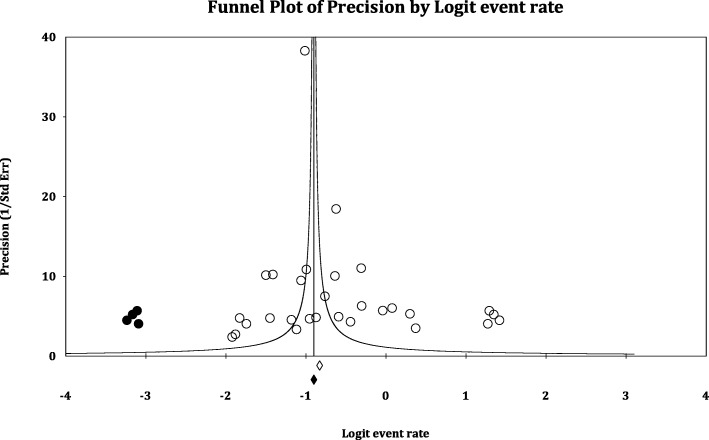
Funnel plot showing found and missing prevalence studies of comorbidities of mental disorders (anxiety and / or depression) and chronic physical diseases (diabetes, obesity, cancers, COPD and heart disease)

The distribution of the pooled studies of the various types of comorbidities was illustrated using a Funnel Plot. From the visual Trim and Fill method of Duval and Tweedie, it was estimated that: there were 4 prevalence studies missing for the comorbidities of mental disorders with chronic physical diseases (Fig. 4) and 3 analytical studies were missing for the comorbidities of mental disorders with chronic physical diseases. The existence of this publication bias could be confirmed with the Egger test with the results: Intercept = 2.85 (*p* = 0.05) and Intercept = 2.75 (*p* = 0.14), respectively, in the prevalence and analytical studies for the comorbidities of mental disorders with chronic physical diseases.

### Sensitivity analysis

Sensitivity analysis after removing the study with the highest weight on the one hand (Lou et al. study [61]) and low quality study on the other hand (Galeano [51]) from the studies included in the pooled prevalence of the comorbidities of mental disorders (anxiety and/or depression) with chronic physical diseases (diabetes, obesity, cancer, COPD and heart disease) varied respectively from 36.6% (95% CI, 31.4–42.1) to 37.0% (30.8–43.6) and 35.9% (95% CI, 30.7–41.4). For the analytical studies, the pooled odds ratio for the comorbidities of mental disorders (anxiety and/or depression) with chronic physical diseases (diabetes, obesity, cancer, COPD and heart disease), which was 3.1 (95% CI, 1.8–5.2), varied to 3.6 (95% CI, 2.0–6.3) when the study with the highest weight (Eslami et al. [49]) was subtracted and 2.6 (95% CI, 1.6–4.2) when low quality studies (Asnaashari et al. [47] and Huang et al. [55]) were subtracted. In general, removing the study with the largest weighting on the one hand and low quality on the other hand had little impact on the results.

### Subgroup analysis

Subgroup analysis to determine the pooled estimates by type of disease investigated, type of associated disease, type of subject included (hospitalised or non-hospitalised) and by study continent gave the following results.

The pooled prevalences in people suffering from chronic diseases were: for depression, 36.4% (95% CI, 28.9–41.6) and for anxiety and/or depression 33.9% (95% CI, 27.1–41.3). A high prevalence of anxiety and/or depression was found: 30.3% (95% CI, 22.5–39.6) for cancer, 41.9% (95% CI, 28.1–57.0) for diabetes, 40.0% (95% CI, 28.7–52.5) for COPD and 50.1% (95% CI, 25.8–74.4) for obesity. If we consider the type of subjects enrolled in the studies, the prevalence of mental disorders in chronic diseases was 39.2% (95% CI, 31.1–47.9) in hospitalised subjects and 34.3% (95% CI, 27.2–42.2) in non-hospitalised subjects. Most of the studies were concentrated in the Asian continent (24 studies), followed by the American continent (3 studies), the African continent (2 studies) and the European continent (1 study). The prevalence of comorbidities of mental disorders with chronic diseases was 45.8% (95% CI, 25.1–68.1) in Africa, 26.8% (95% CI, 14.8–43.5) in America and 37.0% (95% CI, 30.9–43.5) in Asia.

**Fig. 5:**
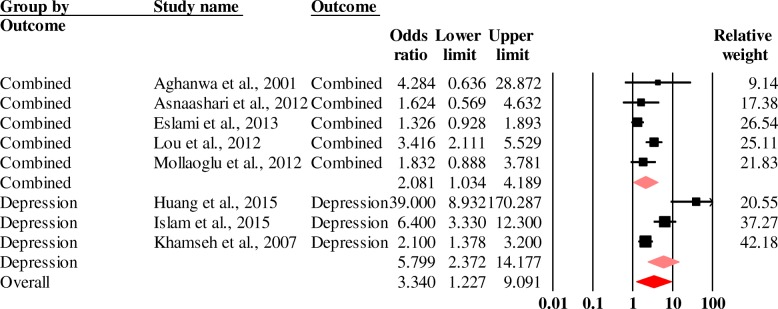
Forest plot of pooled odds ratios of comorbidities of mental disorders and chronic physical diseases by type of mental disorder sought. Heterogeneity: Q = 36.76, df = 7.00, p < 0.0001, I2 = 80.96. Combined = anxiety & Depression

Case–control studies on mental disorders with chronic physical diseases were mostly found on the Asian continent with a statistically significant odds ratio of 3.0 (95% CI, 1.7–5.3). In Africa the association was searched in only one included study [41]. An association of anxiety and/or depression was found in non-hospitalised subjects. By type of disease investigated, the results reveal that the odds ratio of people with chronic physical diseases having depression is 5.8 (95% CI, 2.4–14.2) and the odds ratio of having both depression and anxiety is 2.1 (95% CI 1.0–4.2) (Fig. 5). According to the type of original disease, a significant association of 6.4 was found (95% CI, 1.5–28.0) between obesity and mental disorders (anxiety and depression).

### Meta-regression

The results of the meta-regression about the covariates quality score and type of country obtained by each study are presented below (Table 1). This was only positive for the covariables quality score and income level respectively for the prevalence and analytical studies of comorbidities of mental disorders and chronic physical diseases.

**Table 1:** Meta-regression according to the Country Income Level and the Study Quality Score

Co-morbidity	Covariate	Number of studies	Coefficient	Z	*p*	*T* ^*2*^	*R* ^*2*^
1	Income Level	29	- 0,6286	- 0,89	0,3757	0,3653	0,00
	Quality Score	29	18,492	3,00	0,0027	0,3653	0,10
2	Income Level	8	17,542	3,09	0,0020	0,4177	0,23
	Quality Score	8	1100	0,89	0,3740	0,4177	0,00

1: Comorbidities of mental disorders and chronic physical diseases of prevalence studies

2: Comorbidity of mental disorders and chronic physical diseases of analytical studies

## Discussion

The choice of diseases focused on mental disorders (anxiety, depression, bipolar disorders and schizophrenia) [5, 29] and chronic physical diseases (diabetes, obesity, cancer, heart disease and COPD) [6, 30] because these represent the greatest cause of deaths worldwide.

In our meta-analysis, the diagnosis of the various diseases in the studies included had been performed by acceptable diagnostic methods (Table 1 and Additional file 1: Table S1 and Additional file 2: Table S2). In fact, the standard diagnostic methods used to diagnose mental disorders had been developed in accordance with DSM-5 (Diagnostic and Statistical Manual for Mental Disorders) and ICD-10 (International Classification of Diseases) [73]. However, as regards the diagnosis of mental disorders, the choice of questionnaire may influence the results. In our studies, Gamze Kirkil et al. demonstrated a 54.5% prevalence of depression in patients with chronic obstructive pulmonary disease when they performed the diagnosis with the BDI (Beck Depression Inventory) and 63.8% when the diagnosis had been performed with the HAD-D (Hospital anxiety and depression) [59]. Several other studies point in the same direction [81–84]. Conversely, one study has shown there was no difference in the results between the self-questionnaire and the standardised diagnosis [85]. The diversity of questionnaires found in our studies may be explained by the need to adapt the questionnaire to the population studied [41, 50, 54–56, 66, 68, 78, 80] as is recommended. Only 6.5% of the prevalence studies [60, 63] on the comorbidities of anxiety and/or depression in people with chronic physical diseases (cancer, diabetes, obesity, cardiovascular disease and COPD) used a self-assessment questionnaire such as the Self-rating anxiety scale and the Self-rating depression scale. Contrary to the guide to good laboratory practice and quality assurance for laboratory diagnosis which recommends the use of two different diagnostic tests (one very sensitive and the other very specific), the handling of samples by two different technicians unaware of the subject’s clinical condition, the studies included in our meta-analysis only considered a single, sensitive, specific test. This is acceptable given the constraints associated with the cost of the analyses. For the chronic diseases, in some studies these were confirmed by following diagnostic guidelines [55] or by choosing people whose disease was already known and confirmed in a specialised care facility.

The assessment of study quality demonstrated that the studies we considered for the meta-analysis were of good quality and that our scores are similar to those obtained by Amerio et al. in their meta-analysis [86]. There was publication bias and significant heterogeneity in our estimates. In fact, the different heterogeneity values obtained indicated that the observed heterogeneities were due to some covariates. Thus, with the help of meta-regression we explained these heterogeneities. However, given the null and insignificant *R*^*2*^ values of the meta-regression, it was obvious that there were other covariates which could account for these heterogeneities. For example, the *R*^*2*^ of 0.23 found in the comorbidity of mental disorders and chronic physical diseases in the analytical studies indicates that 23% of the variation found was due to differences in per capita income levels in the 6 countries (China, Iran, Nigeria, Turkey, Bangladesh and Iran) where the 8 included studies were conducted. Despite these attempts at justification, the heterogeneity of the studies included in it remained significant and other covariates could be the reason. In the other types of comorbidities studied, this did not explain the observed variation. Thus, thanks to these results, we can notice that our estimates are not affected by the income level of countries or the quality of studies.

People with mental disorders have a tendency to develop more chronic diseases such as obesity [87] and diabetes [88]. We found a high prevalence, 16.2%, of diabetes and/or obesity in people with schizophrenia or bipolar disorder. Similar results were found in a meta-analysis carried out in Canada with an obesity prevalence of 19% in people with depression. Our result in a hospital setting is also close to the prevalence of type 2 diabetes in schizophrenic patients found in a retrospective study conducted over 6 years in the United States [89] and the prevalence of obesity found in the general Canadian population of 12%. Conversely, our prevalence is less than those of type 2 diabetes, found in 26% of people with bipolar disorders in the United States [89] and obesity, found in 42.1% of patients with schizophrenia in Canada [90]. It is higher than the 6.7% of diabetics found in a study in Belgium in patients with bipolar disorders [91]. These studies show that the prevalence of diabetes metillus is 4 to 5 times higher in people with schizophrenia [92] and that of type 2 diabetes is 3 times higher in the subpopulation of people with bipolar disorders [88] compared to the prevalence in the general population. Despite the reduced number of studies, the significance level of our result conveys the alarming nature of chronic physical diseases for people with mental disorders. It also shows how important and necessary their diagnosis and early management is, especially in developing and emerging countries.

In contrast to the insufficient number of studies found for chronic diseases in people with mental disorders in our meta-analysis, some meta-analyses involving multiple countries in North America, Europe, and Asia [84, 93–96] do have data showing that people with mental disorders have an increased risk of developing chronic physical diseases. This lack of data in developing and emerging countries may be explained by the lack of medical consultations for people with mental disorders and also by the rather reduced number of health centres which, when they exist, are not accessible [97]. In addition, the influence of religion also has a significant impact on the diagnosis of mental disorders in developing and emerging countries. Often wrongly attributed to a spiritual effect, 80% of people with mental disorders and their families prefer to consult religious leaders or healers or exorcist-priests [98]. The lack of financial means also aggravates this situation and is an obstacle to consultation for people who wish to access the appropriate services [99].

Several studies in this meta-analysis considered mental disorders in people with chronic diseases such as cancer, diabetes, obesity, cardiovascular disease and COPD. This is because the policy in developing and emerging countries in this epidemiological transition period favours fighting non-communicable diseases [3]. The prevalence of depression in people with chronic diseases is in line with the results of the meta-analysis by Solano et al. [100] which shows a prevalence of depression of between 13 and 79% in people with chronic physical diseases (cancer, heart disease, renal disease and COPD). The difference in the prevalences for mental disorders comorbidities in people with chronic diseases depends on the “hospitalised” or “non-hospitalised” status of the subject and may be due to their health status. This is often more severe in hospitalised patients for whom there is a higher prevalence (which seems logical). Similarly, Ren et al. [101] in their meta-analysis on depression and coronary heart disease found pooled prevalences of 51% (95% CI, 43–58) for depression in coronary disease in hospitalised patients and prevalences of 34.6 to 45.8% in non-hospitalised patients.

In addition, our meta-analysis revealed that the increased risk for anxiety and/or depression in people with chronic physical diseases was 310% (95% CI, 1.8–5.2). The results of meta-analyses in other countries with lower estimates give odds ratios of: 1.27 to 1.55 for depression associated with obesity in hospital [102–104] and an odds ratio of 1.4 (95% CI, 1.2–1.6) for anxiety associated with obesity [105].

Despite efforts made to minimize bias in our meta-analysis, it still had some limitations, mostly linked to the included studies. These limitations mainly involve: the size of the various samples, which were often relatively small; the diagnosis of mental disorders not being confirmed by a specialist in some studies; and the significant number of studies performed in Asia, which could be an obstacle to generalising the results to all developing and emerging countries. In addition, some estimates could not be determined because of a lack of studies which met our inclusion criteria, especially for chronic diseases comorbidities in people with mental disorders. Mental disorders are also very common during the perinatal period. This is a criterion that could have been included in our inclusion criteria. But the objective of this meta-analysis was oriented towards general results. So, this type of specificity has not been considered. Finally, the fact that we chose to perform our sensitivity test by removing the studies with the greatest weighting could be considered a limitation by those who prefer to subtract the poorer quality studies when performing their sensitivity test. However, it is generally recommended that the weighting of the studies be considered as we have done here.

Nonetheless, our meta-analysis is the first to involve only developing and emerging countries for the comorbidities of mental disorders and chronic physical diseases It was performed following the PRISMA 2015 guidelines and the protocol was registered in the PROSPERO international prospective register of systematic reviews database. Finally, with the performance of the meta-regression, it was possible to show that the quality of studies included in our meta-analysis was above average and did not affect our pooled estimates.

## Conclusion

In conclusion, our pooled estimates show that the comorbidities of mental disorders and chronic physical diseases are indeed a real burden for people in developing and emerging countries. Given the difficulties in developing and emerging countries, prioritisation of the mental health sector is essential in these countries experiencing the increasing burden of non-communicable diseases because there is “No health without mental health” [106]. In addition, awareness of mental health must be integrated into all aspects of health and social policy, into the planning of the healthcare system and into the provision of primary & secondary healthcare. Finally, policies and healthcare for mental health must be reoriented to place more emphasis on the simultaneous diagnosis and treatment of mental disorders and other associated diseases. Through this first meta-analysis in developing and emerging countries, we hope to open the way for other studies, especially cohort studies on comorbidities in mental health. More specifically, studies on subjects with mental disorders in whom chronic physical diseases will be investigated because of the lack of specific data on this comorbidity. In Oceanic, African, and American countries, it is essential to develop research on these co-morbidities.

## Additional files

Additional file 1: **Table S1.** Characteristics of Prevalence Studies of Co-morbidities of Mental Disorders with Chronic Physical Diseases. MINI: Mini International Neuropsychiatric Interview, HADS: Hospital Anxiety and Depression Scale, BDI: Beck Depression Inventory, STAI: State-Trait Anxiety Inventory, PHQ: Patient Health Questionnaire, DSM: Diagnostic and Statistical Manual for Mental Disorders, SCIDI/CV: Structured Clinical Interview for DSM-IV—Clinical Version; PSE: Present State Examination, MMSE: Mini Mental State Examination, SADS-L: Schedule for Affective Disorders and Schizophrenia-Lifetime, MSE: Mental Status Examination, SAS: Self- rating anxiety scale, SDS: Self-rating depression scale, HAMA: Hamilton Anxiety Rating Scale, HAMD: Hamilton Depression Rating Scale, ICD: International Classification of Diseases, HAD-D: Hospital anxiety and depression, FBG: Fasting Blood Glucose; HbA1c: Glycated Hemoglobin, COPD: Chronic Obstructive Pulmonary Disease, CVD: CerebroVascular Disease, F: Female, M: male, Age in years. (DOCX 149 kb)

Additional file 2: **Table S2.** Characteristics of Analytical Studies of Co-morbidities of Mental Disorders with Chronic Physical Diseases. COPD: Chronic Obstructive Pulmonary Disease, GHQ: General Health Questionnaire, PSE: Present State Examination, ICD: International Classification of Diseases, IDF: International Diabetes Federation, SCL: Syndrome Checklist, SCL: Syndrome Checklist, HADS: Hospital Anxiety and Depression Scale, BDI: Beck Depression Inventory, PHQ: Patient Health Questionnaire, BAI: Beck Anxiety Inventory, BAE: Beck Depression Inventory, F: Female, M: male, Age in years. (DOCX 129 kb)

Additional file 3: **Table S3.** Characteristics of quality scores of prevalence studies. Global quality (Items: 1–2–3-5-6-7-9-10); External validity (Items:11–12-13); Results bias (Items: 15–16–18-20); Confusion and selection bias (Item: 25); Power (Item: 27) and S: Quality score. (DOCX 71 kb)

Additional file 4: **Table S4.** Characteristics of quality scores of analytical studies. Global quality (Items: 1–2–3-5-6-7-9-10); External validity (Items:11–12-13); Results bias (Items: 15–16–18-20); Confusion and selection bias (Item: 25); Power (Item: 27) and S: Quality score. (DOCX 30 kb)
