# Supplementary material for: Co-morbidities of mental disorders and chronic physical diseases in developing and emerging countries: a meta-analysis
Source: BMC Public Health. 2019 Mar 13;19:304. doi: 10.1186/s12889-019-6623-6 (PMC6417021; doi:10.1186/s12889-019-6623-6)
Supplement: Supplementary file 1 — Table S1. Characteristics of Prevalence Studies of Co-morbidities of Mental Disorders with Chronic Physical Diseases. MINI: Mini International Neuropsychiatric Interview, HADS: Hospital Anxiety and Depression Scale, BDI: Beck Depression Inventory, STAI: State-Trait Anxiety Inventory, PHQ: Patient Health Questionnaire, DSM: Diagnostic and Statistical Manual for Mental Disorders, SCIDI/CV: Structured Clinical Interview for DSM-IV—Clinical Version; PSE: Present State Examination, MMSE: Mini Mental State Examination, SADS-L: Schedule for Affective Disorders and Schizophrenia-Lifetime, MSE: Mental Status Examination, SAS: Self- rating anxiety scale, SDS: Self-rating depression scale, HAMA: Hamilton Anxiety Rating Scale, HAMD: Hamilton Depression Rating Scale, ICD: International Classification of Diseases, HAD-D: Hospital anxiety and depression, FBG: Fasting Blood Glucose; HbA1c: Glycated Hemoglobin, COPD: Chronic Obstructive Pulmonary Disease, CVD: CerebroVascular Disease, F: Female, M: male, Age in years. (DOCX 149 kb) [file 12889_2019_6623_MOESM1_ESM.docx]

| Reference | Continent | Subjects  type | Original  disease | Associated disease | Positive  (n) | Total  (N) | Diagnostic method | F/M | Average age |
| --- | --- | --- | --- | --- | --- | --- | --- | --- | --- |
| Gomes et al. [52]  Rev Bras Psiquiatr, 2013 | America | Non-hospitalised | Bioplar disorder | Diabeties  Obesity | 21  61 | 159 | – | 107/52 | 43.5±12.0 |
| Sweileh et al. [75]  Diabetes Res Clin Pract, 2013 | Asia | Non-hospitalised | Schizophrenia | Diabeties | 27 | 250 | FBG (Germany)  HbA1c (USA) | 68/182 | 41.9 ±11.8 |
| Maia et al. [63]  Compr Psychiatry, 2012 | America | Non-hospitalised | Diabeties | Anxiety and Depression | 42  18 | 200 | MINI | 124/76 | 59±12.9 |
| Akena et al. [42]  J Affect Disord, 2015 | Africa | Non-hospitalised | Diabeties | Depression | 152 | 437 | MINI | 283/154 | 51±14.06 |
| Al-Amer et al. [45]  J Diabetes Complications, 2011 | Asia | Non-hospitalised | Diabeties | Depression | 128 | 649 | PHQ-8 | 367/282 | 57.34 ±12.08 |
| Zhang et al. [79]  Arch Psychiatr Nurs, 2008 | Asia | Hospitalised | Diabeties | Depression | 100 | 124 | SDS | – | 60.32 |
| Tuncay et al. [77]  Health Qual Life Outcomes, 2008 | Asia | Non-hospitalised | Diabeties | Anxiety | 128 | 161 | Anxiety Scale Turkish version | 98/63 | 49.01±9.74 |
| Galeano [51]  Eureka Assuncion, 2011 | America | Non-hospitalised | Obesity | Depression | 64 | 111 | BDI | – | – |
| Stolic et al. [73]  Indian J Med Sci, 2010 | Europe | Non-hospitalised | Obesity | Depression | 67 | 157 | HAD-D | 109/48 | 56±12.5 |
| Akyol et al. [43]  Jpn J Clin Oncol, 2015 | Asia | Non-hospitalised | Colorectal Cancer | Anxiety and Depression | 30  46 | 105 | HADS- Turkish version | 33/72 | 52.87±9.003 |
| Alacacioglu and al. [44]  Support Care Cancer, 2010 | Asia | Non-hospitalised | Colorectal Cancer | Anxiety and Depression | 26 | 110 | BDI and STAI Turkish version | 45/65 | 58±12.7 |
| Tavoli et al. [76]  BMC Gastroenterology, 2007 | Asia | Hospitalised | Gastrointestinal cancer | Anxiety and Depression | 67  81 | 142 | HADS – Iranian version | 62/80 | 54.1±14.8 |
| Priscilla et al. [71]  East Asian Arch Psychiatry, 2011 | Asia | Hospitalised | Hematological cancer | Anxiety and Depression | 33  26 | 105 | MINI –Version6.0.0 | 55/50 | 40 |
| Pandey et al. [69]  Psychooncology, 2007 | Asia | Non-hospitalised | Head and neck cancer | Anxiety | 17 | 123 | HADS- Malaya-lam Version | 30/93 | 54.2±11.8 |
| Alexander et al. [46]  Acta Oncol Stockh Swed, 1993 | Asia | Hospitalised | Cancer | Depression | 8 | 60 | DSM-111-R | 24/36 | 53.2±13.9 |
| Atesci et al. [48]  Support Care Cancer, 2004 | Asia | Hospitalised | Cancer | Anxiety and Depression | 3  53 | 117 | HADS  SCIDI/CV Turkish version | 60/57 | 53.7±14.2 |
| Hamdan-Mansour et al. [53]  J Cancer Educ, 2015 | Asia | Hospitalised | Cancer | Depression | 72 | 92 | BDI-II | 53/39 | 50.8±15 |
| Li [60]  Asian Pac J Cancer Prev, 2009 | Asia | Non hospitalised | Cancer | Anxiety and Depression | 167  271 | 511 | SAS  SDS | 317/194 | – |

| Reference | Continent | Subjects type | Original  disease | Associated disease | Positive  (n) | Total  (N) | Diagnostic method | F/M | Average age |
| --- | --- | --- | --- | --- | --- | --- | --- | --- | --- |
| Hong et al. [54]  Support Care Cancer, 2014 | Asia | Hospitalised | Cancer | Anxiety and Depression | 79  812 | 1217 | HADS-Chinese version | 490/727 | 51.24±13.06 |
| Zhao et al. [80]  Gen Hosp Psychiatry, 2014 | Asia | Hospitalised | Cancer | Depression | 119 | 460 | MINI 5.0- Chinese version | 234/226 | 59.4± 12.0 |
| Fanger et al. [50]  Rev Assoc Medica Bras, 2010 | America | Hospitalised | Cancer | Depression | 124 | 675 | HADS-Brazilian Version | 252/423 | 56.4±15.4 |
| Maneeton et al. [64]  Asian Pac J Cancer Prev, 2012 | Asia | Hospitalised | Cancer | Depression | 32 | 108 | PHQ-9 | 61/47 | – |
| Karakurt et al. [57]  Int J Nurs Pract, 2013 | Asia | Hospitalised | COPD | Anxiety and Depression | 93  71 | 255 | HADS | 101/154 | 68.87±10.97 |
| Kirkil et al. [59]  Klin Psikofarmakol Bul, 2015 | Asia | Non-hospitalised | COPD | Anxiety and Depression | 22  42 | 80 | BDI  HADS | 17/63 | 68.12±10.25 |
| Lou et al. [61]  Respir Care, 2014 | Asia | Non-hospitalised | COPD | Anxiety and Depression | 2741  1542 | 7787 | HADS | 4062/3725 | 61.7±13.1 |
| Mehta et al. [65]  East Asian Arch Psychiatry, 2014 | Asia | Non-hospitalised | COPD | Anxiety and Depression | 11  19 | 59 | HADS | 5/54 | 60.23± 8.45 |
| Yıldırım et al. [78]  Int J Nurs Pract, 2013 | Asia | Hospitalised | COPD | Anxiety and Depression | 139  171 | 200 | HAD-Turkish Version | 95/105 | – |
| Morsi et al. [67]  Egypt J Chest Dis Tuberc, 2014 | Africa | Hospitalised | COPD | Anxiety and Depression | 28  29 | 48 | HAMA  HAMD | 3/45 | 66.8±7.8 |
| Negi et al. [68]  Indian J Med Res, 2014 | Asia | Non-hospitalised | COPD | Depression | 62 | 126 | PHQ-9-Hindi Version | 33/93 | 62.69±0.84 |
| Rosrita et al. [72]  Pneumol Buchar Rom, 2016 | Asia | Non-hospitalised | COPD | Depression | 27 | 141 | MINI-ICD 10 | 10/130 | – |
| Peltzer et al. [70]  South Afr J Psychiatry, 2016 | Asia | Non-hospitalised | CVD – Diabeties COPD and Cancer | Anxiety and Depression | 355  888 | 1693 | HADS | – | – |
