# Supplementary material for: Co-morbidities of mental disorders and chronic physical diseases in developing and emerging countries: a meta-analysis
Source: BMC Public Health. 2019 Mar 13;19:304. doi: 10.1186/s12889-019-6623-6 (PMC6417021; doi:10.1186/s12889-019-6623-6)
Supplement: Supplementary file 2 — Table S2. Characteristics of Analytical Studies of Co-morbidities of Mental Disorders with Chronic Physical Diseases. COPD: Chronic Obstructive Pulmonary Disease, GHQ: General Health Questionnaire, PSE: Present State Examination, ICD: International Classification of Diseases, IDF: International Diabetes Federation, SCL: Syndrome Checklist, SCL: Syndrome Checklist, HADS: Hospital Anxiety and Depression Scale, BDI: Beck Depression Inventory, PHQ: Patient Health Questionnaire, BAI: Beck Anxiety Inventory, BAE: Beck Depression Inventory, F: Female, M: male, Age in years. (DOCX 129 kb) [file 12889_2019_6623_MOESM2_ESM.docx]

| Reference | Continent | Subjects type | Original disease | Associated disease | Positive  (n) | Total  (N) | OR | Diagnostic method | F/M | Age (Case / Control) |
| --- | --- | --- | --- | --- | --- | --- | --- | --- | --- | --- |
| Lou et al. [62]  BMC Pulm Med, 2012 | Asia | Non-hospitalised | COPD | Anxiety and Depression | – | 1 100  1 100 | 3,26  3,58 | HADS | 546/1654 | 62  (Case & Control) |
| Asnaashari et al. [47]  Iran J Allergy Asthma Immunol, 2012 | Asia | Hospitalised | COPD | Anxiety and Depression | 12 et 13  13 et 11 | 26  33 | – | SCL-90-R, Beck and Hamilton | 32/27 | 57.61 ± 11.80  39.42 ± 6.80 |
| Aghanwa et al. [41]  Journal of Psychosomatic Research, 2001 | Africa | Hospitali Hospitalised | COPD | Anxiety and Depression | 3 et 5  4 et 1 | 30  60 | – | GHQ-30 English or yourouba version; ICD-10 PSE; SCL | 15/90 | – |
| Mollaoglu et al. [66]  Back Musculoskelet Rehabil, 2012 | Asia | Non-hospitalised | Obesity | Anxiety and Depression | 36 et 33  26 et 25 | 60  60 | – | BDI ; BAI & BAE (Turkish Version) | 60/60 | 49±8.8 37.4±6.65 |
| Huang et al. [55]  Horm Athens Greece, 2015 | Asia | Non-hospitalised | Obesity | Depression | 52  2 | 88  56 | – | BDI-II (Chinese Version) | – | 26.25±8.9  24.4±7.6 |
| Islam et al. [56]  J Glob Health, 2015 | Asia | Non-hospitalised | Diabeties | Anxiety and Depression | – | 591  591 | 6,4 | PHQ–9 (Validated on the people of Bangladesh) | 674/508 | 50.4±11.4  (Case & Control) |
| Khamseh et al. [58]  Int J Psychiatry Med, 2007 | Asia | Non-hospitalised | Diabeties | Depression | – | 85  170 | 2,1 | BDI | 239/136 | 47.2±16.3  (Case & Control) |
| Eslami et al. [49]  J Psychosom Res, 2013 | Asia | Hospitalised | Cardiac coronary heart disease | Anxiety and Depression | 62  61 | 347  353 | – | HADS (Persian Version) | 363/337 | 33.2 ± 12.11  33.49 ± 12.18 |
| Subashini et al. [74]  J Postgrad Med, 2011 | Asia | Hospitalised | Schizophrenia | Diabeties | 20  38 | 131  524 | – | American Diabetes Association and IDF criteria | 315/340 | 44±12  (Case & Control) |
