# Supplementary material for: Co-morbidities of mental disorders and chronic physical diseases in developing and emerging countries: a meta-analysis
Source: BMC Public Health. 2019 Mar 13;19:304. doi: 10.1186/s12889-019-6623-6 (PMC6417021; doi:10.1186/s12889-019-6623-6)
Supplement: Supplementary file 4 — Table S4. Characteristics of quality scores of analytical studies. Global quality (Items: 1–2–3-5-6-7-9-10); External validity (Items:11–12-13); Results bias (Items: 15–16–18-20); Confusion and selection bias (Item: 25); Power (Item: 27) and S: Quality score. (DOCX 30 kb) [file 12889_2019_6623_MOESM4_ESM.docx]

| References | Item  *1 2 3 5 6 7 9 10 11 12 13 15 16 18 20 21 22 25 26 27* | | | | | | | | | | | | | | | | | | | | S |
| --- | --- | --- | --- | --- | --- | --- | --- | --- | --- | --- | --- | --- | --- | --- | --- | --- | --- | --- | --- | --- | --- |
| Aghanwaa et al. [41] | 1 | 1 | 1 | 1 | 1 | 1 | 1 | 0 | 1 | 1 | 0 | 0 | 1 | 1 | 1 | 1 | 1 | 0 | 1 | 4 | 19 |
| Asnaashari et al. [47] | 1 | 1 | 1 | 0 | 1 | 1 | 1 | 0 | 0 | 1 | 0 | 0 | 0 | 0 | 1 | 1 | 1 | 0 | 1 | 0 | 11 |
| Eslami et al. [49] | 1 | 1 | 1 | 2 | 1 | 1 | 1 | 1 | 0 | 1 | 0 | 0 | 1 | 1 | 1 | 1 | 1 | 1 | 1 | 2 | 19 |
| Huang et al. [55] | 1 | 1 | 1 | 0 | 1 | 1 | 1 | 1 | 1 | 0 | 0 | 0 | 1 | 1 | 1 | 0 | 0 | 0 | 1 | 0 | 12 |
| Islam et al. [56] | 1 | 1 | 1 | 2 | 1 | 1 | 1 | 1 | 1 | 1 | 0 | 0 | 1 | 1 | 1 | 1 | 1 | 1 | 1 | 1 | 19 |
| Khamseh et al. [58] | 1 | 1 | 0 | 0 | 1 | 1 | 1 | 1 | 0 | 1 | 0 | 0 | 1 | 1 | 1 | 1 | 0 | 1 | 1 | 0 | 13 |
| Lou et al. [62] | 1 | 1 | 1 | 0 | 1 | 1 | 1 | 0 | 1 | 1 | 1 | 0 | 1 | 1 | 1 | 1 | 1 | 0 | 1 | 1 | 16 |
| Mollaoglu et al. [66] | 1 | 1 | 1 | 0 | 1 | 1 | 1 | 1 | 0 | 0 | 0 | 0 | 1 | 1 | 1 | 0 | 0 | 0 | 1 | 1 | 12 |
| Subashini et al. [74] | 1 | 1 | 1 | 0 | 1 | 1 | 1 | 1 | 1 | 1 | 1 | 0 | 1 | 1 | 1 | 1 | 1 | 0 | 1 | 1 | 17 |
